# Co‐expression of diurnal and ultradian rhythms in the plasma metabolome of common voles (*Microtus arvalis*)

**DOI:** 10.1096/fj.202201585R

**Published:** 2023-03-01

**Authors:** Andreas Psomas, Namrata R. Chowdhury, Benita Middleton, Raphaelle Winsky‐Sommerer, Debra J. Skene, Menno P. Gerkema, Daan R. van der Veen

**Affiliations:** ^1^ Chronobiology Section, Faculty of Health and Medical Sciences University of Surrey Guildford UK; ^2^ Sleep Section, Faculty of Health and Medical Sciences University of Surrey Guildford UK; ^3^ Department of Chronobiology Groningen Institute for Evolutionary Life Sciences, University of Groningen Groningen the Netherlands

**Keywords:** behavior, chrononutrition, circadian, feeding, metabolomics, Microtus, ultradian

## Abstract

Metabolic rhythms include rapid, ultradian (hourly) dynamics, but it remains unclear what their relationship to circadian metabolic rhythms is, and what role meal timing plays in coordinating these ultradian rhythms in metabolism. Here, we characterized widespread ultradian rhythms under ad libitum feeding conditions in the plasma metabolome of the vole, the gold standard animal model for behavioral ultradian rhythms, naturally expressing ~2‐h foraging rhythms throughout the day and night. These ultradian metabolite rhythms co‐expressed with diurnal 24‐h rhythms in the same metabolites and did not align with food intake patterns. Specifically, under light–dark entrained conditions we showed twice daily entrainment of phase and period of ultradian behavioral rhythms associated with phase adjustment of the ultradian cycle around the light–dark and dark–light transitions. These ultradian activity patterns also drove an ultradian feeding pattern. We used a unique approach to map this behavioral activity/feeding status to high temporal resolution (every 90 min) measures of plasma metabolite profiles across the 24‐h light–dark cycle. A total of 148 known metabolites were detected in vole plasma. Supervised, discriminant analysis did not group metabolite concentration by feeding status, instead, unsupervised clustering of metabolite time courses revealed clusters of metabolites that exhibited significant ultradian rhythms with periods different from the feeding cycle. Two clusters with dissimilar ultradian dynamics, one lipid‐enriched (period = 3.4 h) and one amino acid‐enriched (period = 4.1 h), both showed co‐expression with diurnal cycles. A third cluster solely comprised of glycerophospholipids (specifically *ether‐linked phosphatidylcholines)* expressed an 11.9 h ultradian rhythm without co‐expressed diurnal rhythmicity. Our findings show coordinated co‐expression of diurnal metabolic rhythms with rapid dynamics in feeding and metabolism. These findings reveal that ultradian rhythms are integral to biological timing of metabolic regulation, and will be important in interpreting the impact of circadian desynchrony and meal timing on metabolic rhythms.

## Graphical abstract

The common vole expresses ~2‐h behavioral/foraging rhythms, which we showed to be synchronized between individuals twice daily at the light and dark transitions, respectively. We mapped real‐time activity/feeding status to high‐resolution (every 90 min) measures of plasma metabolites across the light–dark cycle. Ultradian metabolite rhythms (*N* = 148) were detected, often co‐expressed with 24‐h rhythms. Timeseries clustering revealed three main clusters (out of 10), each expressing different ultradian periods (3.4, 4.1, and 11.9 h), which were enriched for different metabolite classes.

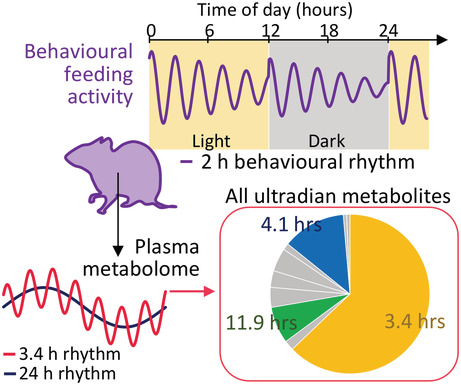

## 1 INTRODUCTION

Circadian metabolic rhythms result from a network of tissue clocks and rhythms generated by cellular, circadian clocks that have evolved in response to the external 24‐h day‐night cycle.^1^ It is overwhelmingly clear that circadian mistiming of sleep and eating, such as experienced by shift workers, can have profound negative health consequences, including obesity and diabetes, directly associated with perturbed circadian rhythmicity of systemic metabolism.^2^, ^3^ On the other hand, time‐restricted eating (intermittent fasting) is rapidly gaining validity as a nutrition program that could have several health benefits including weight loss and improved glucose homeostasis.^4^ Many of the mechanisms underlying negative and positive health outcomes associated with circadian timing of meal patterns remain poorly understood,^5^ and little is known about the rapid dynamics in systemic metabolism associated with meal‐to‐meal patterning.

While understanding mechanisms that drive perturbed circadian metabolic rhythms is deemed paramount by researchers, health professionals, pharmaceutical industry, and funders alike, it is often overlooked that the same metabolic processes also co‐express rapid, <24 h (ultradian) rhythms. Ultradian rhythms are observed in processes such as locomotor activity and feeding behavior, including in rodent species in which the circadian clock has been perturbed.^6^, ^7^, ^8^, ^9^, ^10^, ^11^, ^12^ These cycles are also seen during non‐Rapid‐Eye‐Movement (REM) and REM sleep alternations,^13^ in circulating glucocorticoid levels,^14^ central monoamine release,^15^ and gene expression that is enriched for metabolic processing.^16^, ^17^ These ultradian rhythms have long been hypothesized to maintain metabolic homeostasis,^18^, ^19^, ^20^ and intrinsically driven ultradian rhythms have indeed been observed in various aspects of cellular metabolism that also express circadian rhythms, such as cell mass, ATP concentration, protein synthesis, and respiration in vitro.^21^, ^22^ It is increasingly clear that these ultradian rhythms do not only occur in direct response to food intake and fasting, but are also bona fide biological rhythms; that is, intrinsically driven ultradian rhythms in feeding behavior, gene expression, and cellular metabolism persist in constant conditions including during total fasting in vivo^23^ and in in vitro cell culture.^17^, ^21^, ^22^, ^24^, ^25^

Mechanisms driving these ultradian rhythms remain elusive, but several studies have shown that a reduction in circadian behavioral timing leads to more pronounced expression of ultradian behavioral rhythms,^8^, ^9^, ^10^, ^11^, ^12^ suggesting that drivers of ultradian rhythms have some independence of circadian rhythm drivers. The co‐expression of these circadian and ultradian rhythms in behavior and metabolism requires coordinated “merging” mechanisms that have been shown to favor more prominent expression of ultradian rhythms under conditions of increasing energetic demand.^26^, ^27^, ^28^, ^29^, ^30^ This co‐expression of circadian and ultradian rhythms also suggests that ultradian metabolic dynamics are “masked” by circadian rhythms in most measures gathered in human and animal studies that offer ad libitum intake of high‐quality food. This masking is compounded by the issue that often these studies do not sample metabolism at a high enough temporal resolution (typically only every 4–6 h) to detect ultradian rhythms.

In search of the mechanism(s) that underlie the coordinated co‐expression of ultradian and circadian metabolic rhythms, we turned to the European common vole (*M. arvalis*) which naturally expresses robust ultradian rest/activity and feeding rhythms^31^ associated with its low‐energy fiber diet.^32^ In this laboratory model for ultradian rhythmicity and fiber digestion, we previously showed that their natural ultradian feeding patterns perturb circadian rhythms in the expression of circadian clock genes in the liver, which can be reversed by enforcing a circadian feeding pattern.^18^ Despite this effect of ultradian feeding patterns on circadian clock gene expression in metabolically active tissues, the question remains whether the ultradian feeding patterns are also reflected in systemic metabolism. In this study, we thus aimed to investigate the relationship between diurnal timing and ultradian behavior, and map these ultradian feeding behaviors to the plasma metabolome.

Here, we report that under ad libitum food access conditions, vole behavioral activity associates with ultradian food intake patterns that are synchronized to the light–dark cycle. We show that the vole plasma metabolome expresses ultradian metabolite rhythms that are co‐expressed with 24 h diurnal rhythms in the same metabolites, but do not associate with the naturally expressed ultradian feeding rhythms during ad libitum feeding conditions. Instead, we observed clusters of metabolites that associated with specific metabolite sets which exhibited significant ultradian dynamics with several ultradian periods that were different from the behavioral cycle. These results provide strong evidence for ultradian rhythms in metabolism that do not result from meal patterns, but instead are intrinsically driven.

## 2 MATERIALS AND METHODS

### 2.1 Vole housing and activity measures

Voles were bred in‐house at the University of Surrey, based on founder voles from the colony at the University of Groningen.^18^ All voles were bred and socially housed in cages without running wheels until the start of the experiment. Vole breeding and holding cages were enriched with a running platform (saucer wheel; LBS Biotechnology, Horley, UK) for physical activity, hay for roughage and shelter, carrots for teeth hygiene and nutrient source, and applewood sticks that are critical to appropriate teeth hygiene.^33^ Food and tap‐water were always provided ad libitum. Food included rodent food pellets and forage mix consisting of cereal and dried fruit (LBS Biotechnology). Voles (*N* = 32 males, 32 females) were individually housed at the age of 6 weeks in light‐controlled, sound‐attenuated cabinets at 20–24°C ambient temperature, 55 ± 10% relative humidity. Voles were entrained to 12‐h light, 12‐h dark cycles throughout, and individual cage illumination during the experimental conditions was supplied by LEDs (NSPW500BS, Nichia Europe BV) through frosted glass. The LED spectrum showed a narrow peak at 455 nm and a broader peak at 562 nm^34^ and light intensity at cage bottom was ~170 mW/m^2^ (~50 lux). After 10 days of entrainment, overall behavioral activity was recorded for 10 days using Passive InfraRed (PIR) detectors on the cage lids and total activity was recorded every minute (ClockLab Actimetrics).

The determination of ultradian behavioral activity onsets in the 10‐day activity recordings was performed by fitting a rolling mask (60 min inactivity, 15 min at max activity) to the 1‐min resolution behavioral activity data, and determining the local maximum (in a −75 to 75 min window) of the Least Squared Means Goodness of Fit between behavior and the mask.^35^

Phase response curves were created by depicting XY pairs in which the advance or delay in the timing of the first onset following a change in light conditions (ZT0 and ZT12) is plotted on the Y‐axis, using the number of minutes after the preceding onset at which the stimulus at ZT0 or ZT12 fell on the X‐axis value. The advance or delay in onset time was calculated as the difference between the timing of the first onset of activity after a light transition and the predicted onset calculated to fall exactly one average ultradian period length (132 min) after the preceding onset. Only those pairs of before‐after light transition onsets were included in which the new onset fell within 1 h either side of the behavioral period (thus between 72 and 192 min after the first onset), and light transition occurred between the first and second onset. As negative controls, phase response curves were also calculated for onset pairs around ZT6 and ZT18; timepoints at which no light transitions occurred.

**FIGURE 1:**
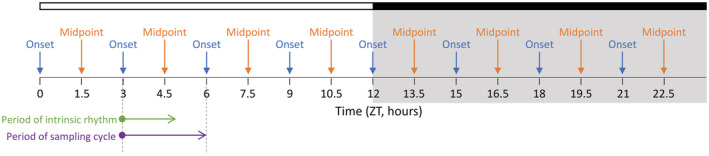
Experimental design to achieve a high temporal resolution mapping of behavioral state to metabolic physiology. This design allowed exact mapping of ultradian behavior to metabolic status on an artificial, 3‐h sampling schedule (period length illustrated by purple arrow), which is dissociated from the intrinsically‐driven 2‐h behavioral cycle (period length illustrated by green arrow). A 3‐h sampling of feeding status was applied by collecting blood at alternating ultradian behavioral onsets and midpoints. Detection of activity for 10 days informed a real‐time algorithm to predict, and then confirm that a behavioral ultradian cycle onset (blue arrow) or resting midpoint (orange arrow) fell within 10 min of a known diurnal timepoint. Voles (*N* = 64, 2 males, 2 females per time point) were sacrificed at each of these diurnal timepoints across the 12 h light/12 h dark (gray areas) cycle, and blood was collected.

To allow precise linking of the natural behavioral activity/feeding pattern to physiological measures, we used a novel design to sample at alternating behaviorally active/fed and the inactive/fasted state every 90 min, which dissociates this 3‐h sampling of feeding status from the intrinsically‐driven 2‐h ultradian rhythm. The fasted state resulted from a natural end of the feeding state under ad libitum food availability, and was tracked and confirmed using real‐time behavioral analysis. Ultradian behavioral onsets were predicted to occur at 1 ultradian cycle after a known onset, and midpoints at 0.5 ultradian cycles from the known onset. If an ultradian onset or midpoint was forecast to fall within 10 min of a diurnal sampling point (every 90 min from ZT0, see Figure 1), the activity of the voles was actively monitored. Real‐time confirmation of an onset at the forecast time required that the vole exhibited complete inactivity for at least 60 min, and when activity occurred it needed to exceed 75% of the average activity for 5 min. If these criteria were met, the vole was sacrificed at those 5 min after behavioral onset. To standardize the acute effects of behavior, only “inactive” midpoints were accepted for sampling which was defined as total inactivity in the preceding 5 min.

All experimental procedures were approved by the University of Surrey Animal Welfare Ethical Review Body and carried out under UK Home Office Project and Personal Licenses.

### 2.2 Correlation and concordance between behavioral activity and eating

At the University of Groningen, a second cohort of male, adult voles (N = 8) were individually housed in cages equipped with a running wheel, food hopper, and PIR detector. Food pellets and tap water were available ad libitum, and voles were entrained to LD 12:12 for 2 weeks, after which the running wheels were manually blocked at ZT6 (middle of the light period) for 5 days, after which the wheel was unblocked. After 48 h following blocking and unblocking, locomotor activity and feeding behavior were recorded for 3 days at 2‐min resolution for each vole using both PIR and food hopper swing. In the case of the unblocked wheel condition, wheel revolutions were also recorded. Data were smoothed using boxcar smoothing (three datapoint window), and Pearson correlations between behavioral measures were established. In addition, concordance between behaviors was defined as the percentage of datapoints in which two behavioral measures were either both active or both inactive.

All animal experiments at the University of Groningen were approved by the Animal Experimentation Committee of the University of Groningen (DEC).

### 2.3 Targeted metabolomics

Voles (*N* = 64) were sacrificed in light during the light phase (ZT0‐12), and under dim red light in dark phase (ZT12‐24). The selection of voles assigned to each timepoint was based on their natural coincidence of ultradian behavioral phase (ultradian onset or midpoint) and diurnal time. Blood samples were collected directly from the heart using cardiac puncture into lithium‐heparin tubes. Blood was centrifuged at 1620× *g* for 10 min at 4°C to separate the plasma from red blood cells, and plasma was stored at −80°C until further processing. Metabolite concentrations (μM) were determined using the AbsoluteIDQ® p180 targeted metabolomics kit (Biocrates Life Sciences) analyzed in‐house on a Waters Xevo TQ‐S mass spectrometer coupled to an Acquity HPLC system.^3^, ^36^, ^37^ Metabolite concentrations were established using either LC/MS (liquid chromatography/mass spectrometry) or FIA/MS (flow injection analysis). Amino acids and biogenic amines analysis was performed by UPLC (Ultra‐performance liquid chromatography) using a reversed phase C18 column and detected by a tandem mass spectroscopy method (UPLC‐MS/MS). Acylcarnitines, glycerophospholipids, and sphingolipids were analyzed by flow injection analysis (FIA) using tandem mass spectroscopy (FIA‐MS/MS). Flow injection analysis used an LC autosampler, injector, and mobile phases to introduce samples into the ion source of a mass spectrometer. Unlike UPLC, with FIA there was no metabolite separation on the analytical LC column before MS/MS detection.

Metabolite detection was achieved with a Waters Xevo TQ‐S triple quadrupole mass spectrometer (MS/MS) operated in Multiple Reaction Monitoring (MRM) mode. Both UPLC‐MS/MS and FIA‐MS/MS used electrospray ionization (ESI) in positive ion mode. The range of MRM transitions and collision energies was provided by Biocrates Life Sciences, manufacturer of the AbsoluteIDQ® p180 targeted metabolomics kit we used. The specific precursor to product ion transition was measured for every metabolite and stable isotope labeled internal standard. Amino acids and biogenic amines were quantified by calibration curves built using seven external standards and internal standard ratios. A single‐point calibration curve was used for relative quantification of the acylcarnitines, glycerophospholipids, and sphingolipids.

Values excluded from analysis included values below the limit of detection or lower limit of quantification, above the limit of quantification, blank out of range or had a coefficient of variation larger than 30% for the internal quality control (QC2). In addition, only metabolites with values in >60% of samples were included. As a result, 148 metabolites (of 183 included in the assay) from five different compound classes were included in the analysis. Data were expressed as Log_10_ concentration and pareto scaled. Principle Component Analysis (PCA; SIMCA v. 13.0; Umetrics) identified four samples with a Hoteling's t value above the 95% confidence interval (1 Female at ZT9, 1 male at ZT16.5 and 2 females at ZT19.5), which were thus excluded, resulting in *N* = 60 samples in subsequent analysis of metabolite time courses. Orthogonal Projections to Latent Structures Discriminant Analysis (OPLS‐DA; SIMCA v. 13.0; Umetrics) was used to test for the classes “behavioral phase” (onset, midpoint of activity) and “sex” (M/F).

### 2.4 Time series analysis

Metabolite time courses were z‐scored using the mean and standard deviation for each individual metabolite, and then clustered into 10 unequally sized clusters based on time course similarity using unbiased hierarchical agglomerative clustering employing correlation as the distance metric. We checked for the presence of diurnal rhythmicity in each cluster using cosinor analysis on all individual z‐scored metabolites concentrations in a cluster combined, treating measures of the different metabolites within a cluster as replicates for each timepoint. For cosinor analysis, we used least square curve fitting (lsqcurvefit; MATLAB, MathWorks) to fit both a sloping line, and a sloping line + cosine model to our data, where the period of the cosine was fixed to 24 h. Using an added‐sum‐of‐square F‐test, we then tested the hypothesis that this line+cosine model was a better fit than the null hypothesis of only a sloping line (*α* = 0.05). The list of p‐values for all clusters was FDR corrected to correct for multiple comparison. If there was no significant diurnal cosine model, the modeling was repeated, but now the period was allowed to self‐determine within the range from 0 to 24 h. To account for local minima during period self‐determination, we re‐ran the fitting of the ultradian model with different initial periods (1–24 h, step size 1.5 h), and accepted the significant ultradian model with the highest *R* ^2^ goodness of fit.

For the detection of a second rhythm that was co‐expressed with significant diurnal or ultradian rhythms, we added a second cosine model to the primary model which was line+cosine, and the period was again allowed to self‐determine within the range of 0–24 h. A second added‐sum‐of‐square F‐test was then used to test whether a second cosine significantly added to the model. All p values were FDR corrected for multiple comparison, using the Benjamini and Hochberg procedure (mafdr using “BHFDR” flag; MATLAB, MathWorks).

## 3 RESULTS

### 3.1 Twice daily resetting of phase and period of ultradian behavioral rhythms

**FIGURE 2:**
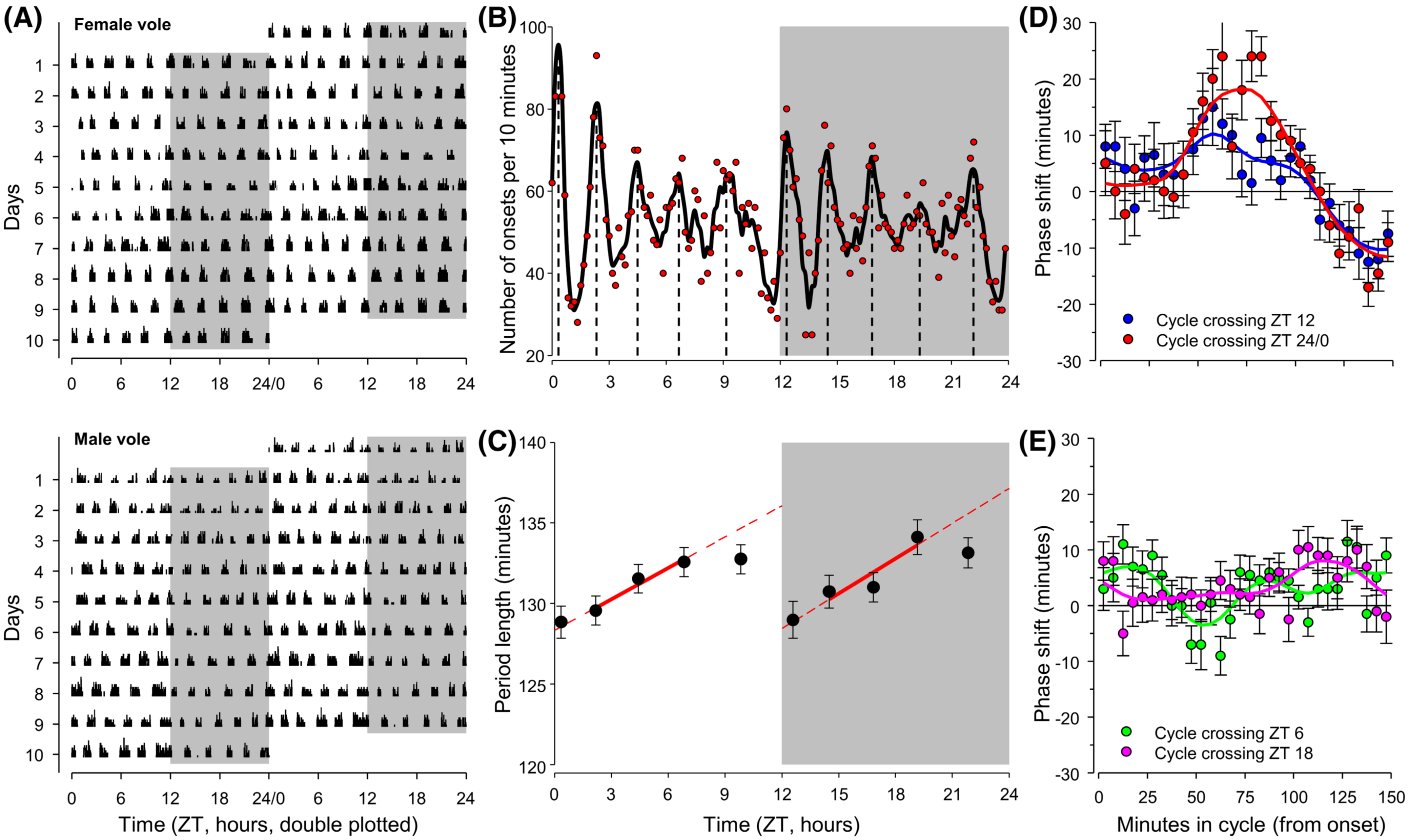
Characteristics of ultradian behavioral activity cycles in the Common vole (*M. arvalis*). (A) Representative plot of 10 days of activity recordings of an individually housed female (top) and male (bottom) vole which were entrained to a 12‐h light/12‐h dark (gray areas) cycle, while all other environmental conditions remained unchanged. Black vertical bars represent relative intensity of overall activity measured using Passive Infrared Recording; each horizontal line represents a single day, data are double plotted on time axis. (B) Histogram of number of behavioral onsets per 10‐min interval (red symbols, total onsets = 7652 onsets of 64 voles over 10 days combined). Black line indicates rolling mean. (C) Mean period length of onset‐to‐onset behavioral cycles in each of the 10 ultradian cycles identified in panel B. Red lines is linear regression through period over the light or dark period based on cycles that do not cross the light or dark transitions; dashed red lines extend linear regression to cycles not included in the linear regression. (D) Phase Response Curves showing advances and delays in behavioral onset timing of the first onset after lights on (ZT24/0, red line and circles) and lights off (ZT12, blue line and circles), as a function of the time in the behavioral cycle at which the change in light conditions occurred. (E) Negative control Phase Response Curves showing advances and delays in behavioral onset timing after non‐existent light/dark transitions at the mid light (ZT6) and mid dark (ZT18).

We entrained 64 adult voles (8 weeks old; 32 males, 32 females) to a 12:12 h light–dark cycle with ad libitum food access and subsequently monitored their overall behavioral activity using passive infrared detectors for 10 days. All male and female voles exhibited strong ultradian behavioral rhythmicity comprising of 10–11 ultradian activity‐rest cycles every 24‐h day (Figure 2A). Using an automated algorithm to detect ultradian onsets of behavioral activity, we identified a total of 7652 ultradian onsets in the 10‐day overall behavioral activity recordings of these 64 voles. A histogram of timing of the occurrences of the behavioral onsets in the voles (Figure 2B) showed that these timings of onsets were synchronized, exhibiting 10 peaks in onset timing spaced across the 24‐h diurnal cycle. The 10 onset‐to‐onset ultradian cycles differ slightly in length across the day (Figure 1C) with periods ranging from 129 ± 1 min (mean ± SEM) to 134 ± 1 min over the 10 cycles. Ultradian periods were shortest immediately after both the light–dark (LD) transition and dark–light (DL) transition, and period length increased during both light and dark episodes, apart from the last cycle before the LD and DL transitions (Figure 2C). When excluding cycles just prior and after the LD and DL transitions, the average ultradian cycle length was 132 min which is in line with previous reports.^18^, ^31^

Following the increase in synchronized occurrences of ultradian behavioral onsets after the LD and DL transitions, onset timings became progressively less synchronized across and between voles, and the distribution of onsets in the populations was best described as a dampening rhythmic cosine wave (*R* ^2^ = 0.639, *p* < .05 against three other models, Figure S1). The relatively shorter period of the last ultradian cycle starting prior to the LD and DL transitions, and the increased synchronization of the subsequent onset after the LD and DL transitions suggests that synchronization of onsets results from a phase adjustment of the ultradian cycle around the LD and DL transitions. We, therefore, constructed Phase Response Curves (PRC) depicting the advance or delay in the timing of the first onset after LD and DL transitions as compared to a predicted onset occurring 132 min (the mean ultradian period of cycles that do not cross LD or DL transitions) after the last onset prior the LD or DL transition. Both the LD and DL PRCs (Figure 2D) exhibit a lack of phase shifting when the LD or DL transition occurs soon after the onset (known as a ‘dead zone’ of the PRC), followed by an advance and subsequent delay zone. As a negative control, we also constructed PRCs for non‐existent light/dark transitions at mid‐light (ZT6) and mid‐dark (ZT18), and these negative control PRCs show no clear advances or delays in behavioral onsets (Figure 2E). Overall, these data indicated that ultradian behavioral timing is entrained to diurnal timing by synchronizing the ultradian behavioral onsets at LD and DL transitions.

### 3.2 Ultradian behavioral activity cycles align with feeding activity

**FIGURE 3:**
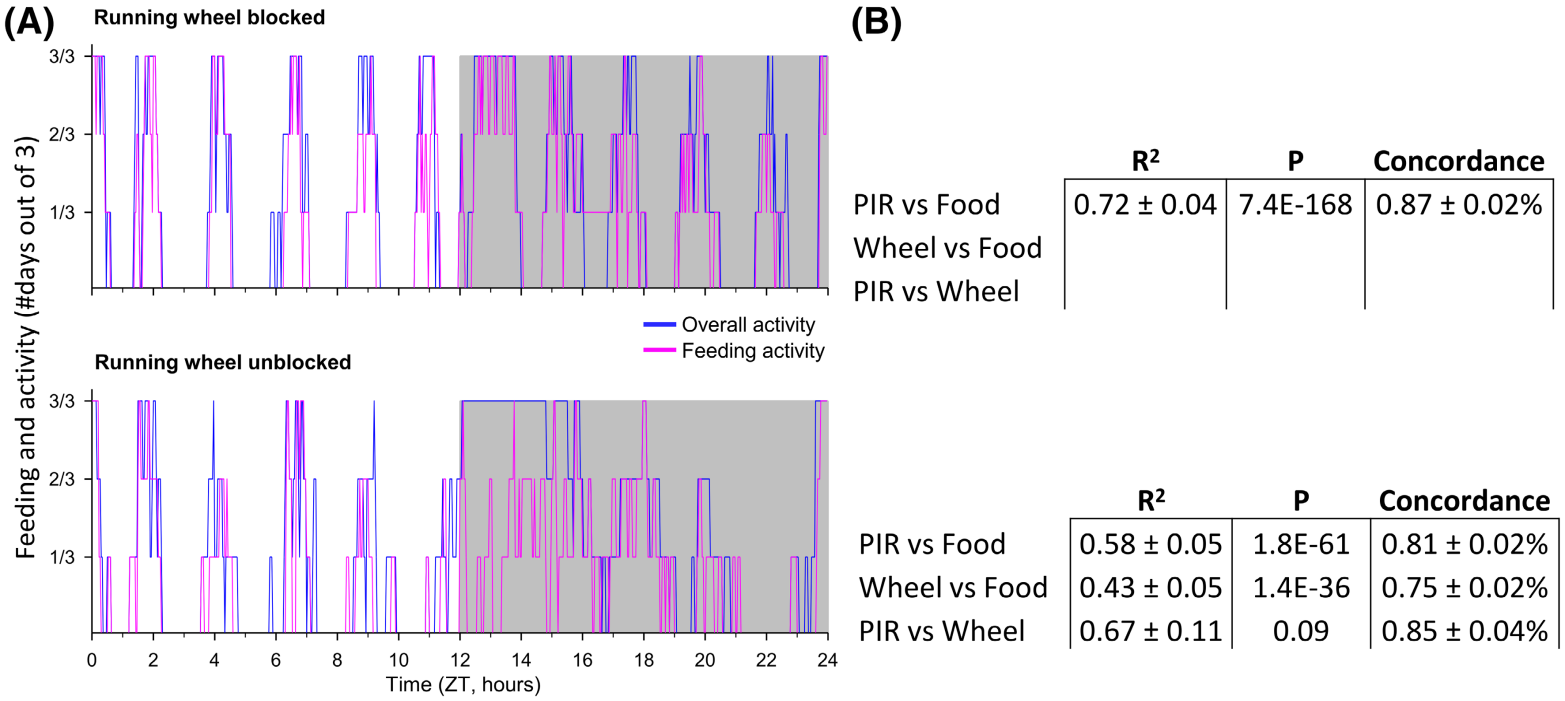
Concordance between overall behavioral activity and feeding behavior at food hopper. (A) line plots of overall activity (blue line) and feeding behavior (pink line) of an example vole housed with a blocked running wheel (upper panel; low levels of circadian organization of behavior) or unblocked running wheel (lower panel; higher levels of circadian organization of behavior). Behavioral state is given on the y‐axis by values indicating that the voles engaged in activity or feeding in 1, 2, or 3 of the 3 consecutive days included in the analysis. (B) Means ± SEM of *R* ^2^ and *p* values of Pearson correlations between each behavioral measure for *N* = 8 male voles. In addition, mean ± SEM of concordance between behaviors is given, which is defined as the percentage of 2‐min time bins in a day in which two behavioral measures were either both active or inactive.

In our experiment, voles were given ad libitum food access to allow expression of natural feeding activity. We sought to confirm that under these conditions voles exhibited feeding activity during active wakefulness, and did not feed during behavioral inactivity. We, therefore, acquired simultaneous recording of rest/activity and feeding activity in a separate cohort of eight male voles housed with blocked running wheels. We confirmed that there was an 87% (±2% SEM) concordance between feeding activity and overall behavioral activity in ultradian voles (Pearson correlation, *P* < 1e^−167^; Figure 3A,B upper panels). The direct link between active wakefulness and feeding was further confirmed by a sustained high concordance between overall behavioral activity and eating activity of 81% (±2% SEM; Pearson *P* < 1e^−61^; Figure 3 lower panels) when the running wheel was unblocked to promote a co‐expressed circadian activity and feeding pattern.^6^, ^18^, ^38^

### 3.3 Co‐expression of diurnal and ultradian rhythms in the vole plasma metabolome

We next tested the hypothesis that natural ultradian behavioral activity or feeding cycles expressed under ad libitum feeding conditions were associated with ultradian dynamics in the plasma metabolome. To test this, we collected a high temporal resolution time series of vole blood samples which dissociates a 3‐h feeding cycle resulting from alternate sampling of natural occurring feeding and fasting states every 90 min from the intrinsically driven 2‐h ultradian behavioral rhythm. Total blood was collected from four voles (2 males, 2 females with ad libitum food access) every 90 min (total N = 64 samples) at alternating ultradian behavioral onsets (end of non‐feeding state) and midpoints (end of feeding state; Figure 3, see methods for real‐time behavioral confirmation of ultradian phases). We performed plasma metabolic profiling using our established in‐house, targeted metabolomics approach,^3^, ^36^, ^37^ and quantified concentration time courses of a panel of 148 known metabolites. Principle Component Analysis identified four samples with a Hotellings t value above the 95% confidence interval, which was removed from further analysis resulting in *N* = 60 samples included in subsequent analyses.

**FIGURE 4:**
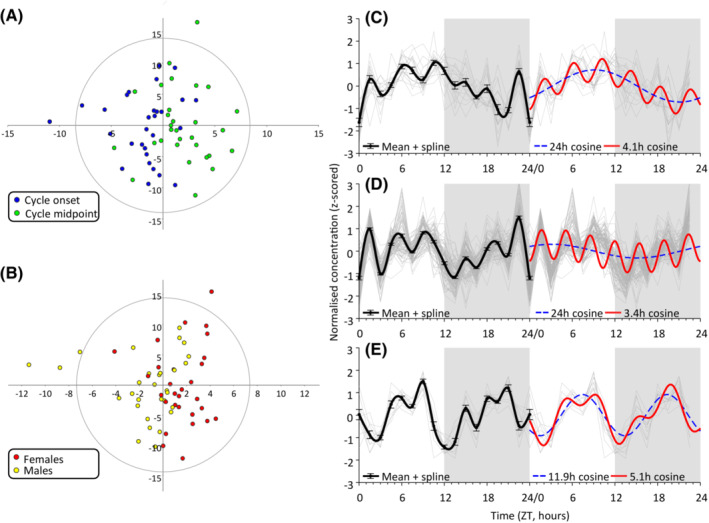
Ultradian and diurnal dynamics in plasma metabolomics in the vole (N = 60; four samples excluded as outlier based on Hotelling's 95% criterion in Principle Component Analysis). OPLS‐DA (SIMCA v. 13.0; Umetrics, Sweden) analysis exhibits only partial separation of metabolomics concentrations for (A) behavioral state (cumulative Q2 = 0.115) and (B) sex (cumulative Q2 = −0.225), indicating that neither behavioral state nor sex were substantially driving metabolite concentration. X‐axis depicts the predictive component (between‐group variation) and Y‐axis depicts the orthogonal component (within‐group variation). Time courses of z‐scored concentration of all metabolites (light gray lines) in the three largest clusters: cluster I (C; *N* = 19), cluster II (D; *N* = 93), and clusters V (E; *N* = 11). Time courses are double plotted on the x‐axis. Black lines indicated spline curves through the cluster mean on the left 24 h. Significant cosine models (*p*'s < 0.05) calculated on single 24‐h time courses are indicated on the right as blue dashed and solid red for the first and second model, respectively.

We tested the hypothesis that metabolite concentrations differed between samples taken at fasted and fed states that result from the behavioral ultradian feeding cycle using discriminant analysis. We observed only partial separation for sampling time phase (Figure 4A; behavioral ultradian onset vs midpoint, OPLS‐DA cumulative Q2 = 0.115; cumulative R2X = 0.509, cumulative R2Y = 0.3) or sex (Figure 4B; OPLS‐DA cumulative Q2 = −0.225, cumulative R2X = 0.497, cumulative R2Y = 0.24), indicating that neither factor was substantially driving metabolite concentration. The lack of substantial separation in the metabolome associated with behavioral state suggests that feeding status does not acutely impose ultradian cycles on the plasma metabolite profile.

Given that metabolite concentrations did not differ between feeding status, we next looked for other prevalent dynamics in metabolite concentrations over time. Using unsupervised clustering of z‐scored metabolite concentrations, we identified groups of metabolite time courses based on similarity. Subsequently, cosine fitting was applied to investigate whether the concentration time course of metabolites in each cluster exhibited rhythmicity, first with the period fixed to 24 h and, if not significant, allowing the period to self‐determine to periods shorter than 24 h (Table 1, First model). We observed that metabolites in four clusters (I–IV in Table 1; total *N* = 125 metabolites (85%)) exhibited a significant 24‐h diurnal pattern (FDR‐corrected Ps <0.05), with acrophases in the light (I, II) and dark (III, IV) phase of the LD cycle. Three other clusters (V–VII in Table 1; *N* = 18 (12%)) showed significant ultradian rhythmicity (FDR‐corrected *p*s < .05) with periods of 11.9, 4.0, and 15.2 h, respectively, with first acrophases of the day falling between 3.5 and 8.1 h. The remaining three clusters (VIII–X) did not display any significant cosine fit (Table 1, no significant model). Given that diurnal and ultradian periods can be co‐expressed, we attempted to add a second cosine model in those clusters that exhibited a diurnal or ultradian rhythm (Models I–VII in Table 1). Significantly co‐expressed ultradian rhythms with periods that differed from the first model were observed in seven clusters, with periods ranging from 3 to 5.3 h (Models I–VII in Table 1, Second model), and first acrophases of the day fell between 1.7 and 4.7 h. The time courses of metabolites in the three largest clusters are displayed in Figure 4 (panels C–E), showing both the mean time course (left, black line) and cosine models (right, red, and blue lines for the first and second cosine models, respectively).

**TABLE 1:** Diurnal and ultradian periods and acrophase in each of the 10 metabolite clusters resulting from significant cosine fitting (FDR‐corrected *p* < 0.05).

Cluster	*N*	First model						Second model		
		Diurnal fit			Ultradian fit			Ultradian fit		
		FDR	Period	Acrophase	FDR	Period	Acrophase	FDR	Period	Acrophase
I	19	0.0E+00	24 h	9.1 h				1.4E‐15	4.1 h	2.0 h
II	93	5.6E‐16	24 h	3.1 h				0.0E+00	3.4 h	1.7 h
III	7	1.6E‐02	24 h	16.5 h				5.5E‐07	2.4 h	1.9 h
IV	6	4.2E‐02	24 h	21.6 h				8.6E‐07	3 h	2.3 h
V	11				0.0E+00	11.9 h	7.4 h	1.5E‐09	5.1 h	4.7 h
VI	5				8.2E‐04	4.0 h	3.5 h	1.2E‐05	3.3 h	1.9 h
VII	2				4.2E‐03	15.2 h	8.1 h	2.1E‐02	5.3 h	1.7 h
										
VIII	3	No significant model								
IX	1	No significant model								
X	1	No significant model								

### 3.4 Metabolite composition differs between ultradian clusters

**FIGURE 5:**
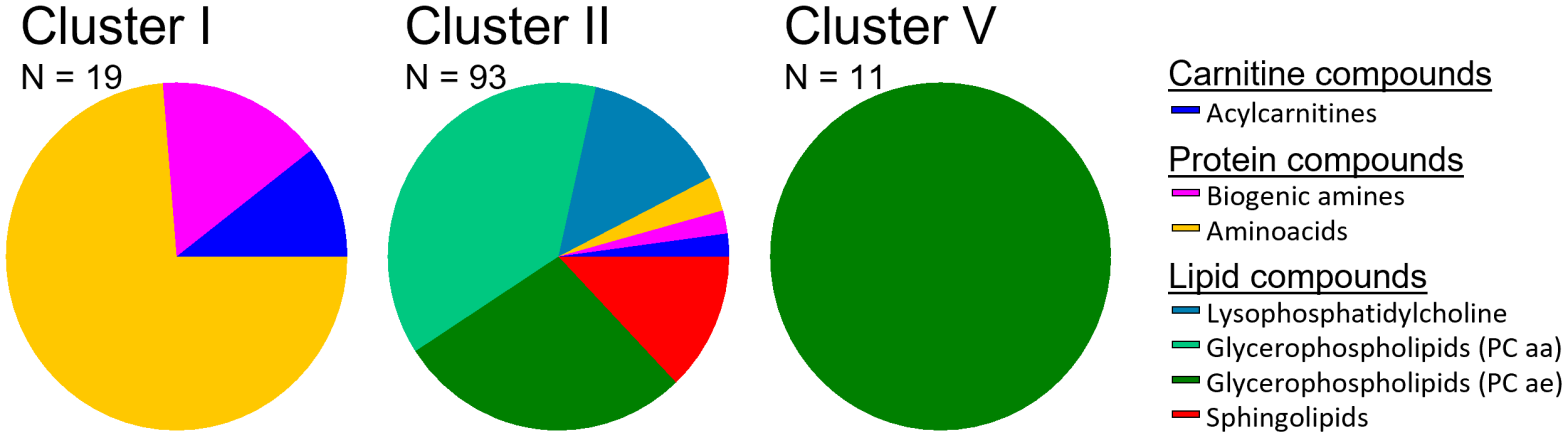
Metabolite composition of the three largest metabolite clusters (I, II, and V).

A hallmark of ultradian rhythms is that they often present with different periods, and their underlying mechanisms (e.g., intrinsic clocks, hour‐glasses, or other mechanisms) remain unknown. We, therefore, investigated whether the observed metabolite cluster with different ultradian periods was associated with specific, but different, metabolic processes. Given that the number of metabolites per cluster were too small to apply meaningful enrichment or pathway analysis, we looked at the contribution of specific metabolite classes in the three largest clusters (Figure 5, Table S1).

Cluster I (Figure 5), which exhibited co‐expressed diurnal and 4.1 h ultradian rhythmicity, encompasses 14 of the 21 detected amino acids (67%), three biogenic amines (methionine‐sulfoxide, sarcosine, and trans‐4‐Hydroxyproline) and two acylcarnitines (carnitine and propionylcarnitine). Cluster I did not contain any lipid compounds. Cluster II (Figure 5B) is the largest cluster and exhibits co‐expressed diurnal and 3.4 h ultradian rhythms and comprises most of the detected lipid compounds, including all detected lysophosphatidylcholines (*N* = 13), all detected phosphatidylcholine diacyls (PC aa, *N* = 35), all detected sphingolipids (*N* = 13), as well as 26 (70%) of the detected phosphatidylcholine acyl‐akyl (PC ae). In addition, cluster II encompasses three amino acids (leucine, phenylalanine, tryptophan), two acylcarnitines (hydroxypropionylcarnitine, propenylcarnitine), and two biogenic amines (kynurenine, serotonin). Cluster V (Figure 5C), which expresses two ultradian rhythms of 11.9 and 5.1 h, exclusively encompassed 11 (30%) PC ae metabolites which were all ether‐linked phosphatidylcholines.

## 4 DISCUSSION

Since Jürgen Aschoff's initial hypothesis that ultradian rhythmicity serves self‐preservation through maintaining metabolic homeostasis,^19^, ^39^ evidence has been accumulating that ultradian metabolic rhythms are co‐expressed with circadian rhythms, but their interactions remained unclear. Using targeted metabolomics, the current study shows ultradian rhythmicity in the vole plasma metabolome which is co‐expressed with diurnal rhythms. Our protocol, which maps metabolite concentration at alternating behavioral phases that reflect feeding and fasting in the vole, does not indicate a strong association between feeding status and metabolite concentration. Instead, ultradian metabolite rhythms are expressed with 3.4, 4.1, and 11.9 h periods. The observation that different metabolites cluster into ultradian rhythms with different periodicities offers mechanistic insights into how several ultradian periods can be co‐expressed with diurnal rhythms in the same organism.^16^, ^17^, ^40^ Our findings in the vole showing coordinated co‐expression of ultradian and diurnal rhythms in feeding behavior and metabolism are relevant to the unknown mechanisms that link energy homeostasis and ultradian biological rhythmicity as observed in several different species,^26^, ^27^, ^28^, ^29^, ^30^ and emphasize the need for adopting high‐resolution sampling of metabolic physiology.

### 4.1 Ultradian behavioral cycles are phase‐entrained twice daily to diurnal timing

Our data show that vole ultradian behavioral cycles are phase‐aligned within‐ and between voles twice a day, which is mediated by phase adjustments at the times of light–dark transitions. The observed phase adjustments that achieve this daily ‘entrainment’ of ultradian rhythms describe ultradian phase response curves around light–dark and dark–light transitions that are reminiscent of circadian phase response curves. Intriguingly, phase re‐adjustments associate with a shortening of the ultradian period, which then progressively lengthens until the next phase re‐adjustment. These shortenings of the ultradian period suggest that the velocity of the underlying mechanisms driving ultradian behavioral cycles are affected by phase adjustments, which is in line with the hypothesis that ultradian behavior is driven by true oscillators.

In our protocol, it remains unclear whether ultradian behavioral entrainment is achieved in response to the light–dark cycle, or results from a direct interaction between ultradian and circadian clocks as has been previously suggested in voles housed under long and short photoperiod.^41^ A direct, twice daily interaction between the circadian and ultradian timing systems that we observed in the highly ultradian vole leads to the hypothesis that this intersection presents as a 12‐h cycle that is not visible during behavioral quiescence in other species such as mice and humans that are strongly circadian, while it may still be expressed in physiological processes. Such 12‐h physiological rhythms could associate with the established 12‐h rhythmicity in the mouse transcriptome,^16^, ^17^, ^42^ the 12‐h metabolic rhythms observed in *Drosophila* ^43^ and in the current study in voles.

### 4.2 Ultradian behavioral cycles associated with food intake

Our simultaneous measures of rest/activity and feeding behavior indicated a high concordance between both behaviors, showing that voles almost exclusively ate during active wakefulness, and fasted during the behavioral quiescence. It is well established that the timing of feeding can act as a potent timing cue for peripheral circadian clocks through direct transcriptional control,^44^, ^45^, ^46^ and we have previously shown that a dominant ultradian feeding pattern quenched the circadian liver clock in the vole, whereas weak ultradian feeding did not.^18^ In the vole, strong ultradian feeding cycles are associated with a high motivation for ingestion due to the low energy yield from its fiber diet.^32^ In several other species, an unfavorable energy intake/expenditure ratio has also been associated with a dynamic increase in ultradian organization of behavior.^26^, ^27^, ^28^, ^29^, ^30^ A dynamic co‐regulation between ultradian and circadian rhythms in metabolism is also reminiscent of ultradian sleep–wake and feeding behavior in human infants in the first weeks and months of life that appears intrinsically driven,^47^ but over time gradually merges with a developing circadian timing system.^48^, ^49^ Taken together, this evidence strongly suggests that an unfavorable energy intake/expenditure ratio promotes ultradian feeding, which in turn reduces the strength of circadian organization of metabolism, unmasking ultradian metabolic dynamics.

### 4.3 Ultradian metabolic rhythms with ~3 to 4 h periods do not map to feeding pattern

We observed two ultradian rhythms in the metabolite panel that were expressed with periods of 4.1 h (*N* = 19) and 3.4 h (*N* = 93), both of which were co‐expressed with diurnal rhythms. These clusters were enriched for amino acids and lipids, respectively. Both ultradian periods were different from the 3‐h sampling of fed and fasted states associated with feeding cycles. Discriminant analysis confirmed that metabolite concentrations do not separate on behavioral state. These ultradian metabolite rhythms were thus unlikely to occur as post‐prandial excursions in circulating metabolite concentrations, but are hypothesized to be intrinsically driven instead. It is not clear whether the difference in ultradian periods between the two clusters is indicative that these rhythms were driven by different mechanisms, or whether the observed 0.7 h difference in period length could result from the different biochemistry of these clusters (lipids vs. amino acids) but were driven by the same underlying mechanisms. Either way, to our knowledge there are no known mechanisms (clocks, hourglass, or otherwise) that could drive such endogenous metabolite rhythms.

Our protocol was designed to separate the direct effects of feeding state (sampled as a 3‐h rhythm) from the intrinsic ~2‐h behavioral rhythm. While the observed rhythms do not map to feeding state, it is conceivable that these 3–4 h metabolite rhythms are detected with a period that is a multiple of the intrinsic ~2 h behavioral rhythm, which could only be detected if we increased our sampling resolution further. For this reason, cosinor fitting was chosen for period analysis, a method that sits in the time domain rather than the frequency domain, and is thus less sensitive to harmonics. In our cosine fitting approach, we could also fit a single cosine to 6–7 ultradian cycles, allowing the model to be fitted to all metabolite time courses in a cluster at once, treating each metabolite as a “replicate,” and adding robustness to the fit against noise.

The observation of not one, but multiple, ultradian periods is in line with other reports^16^, ^17^, ^42^ that suggest that several groups of ultradian rhythms present with different periods, all of which allow several full ultradian cycles to be completed in a single circadian/diurnal cycle (24/*N* periods; i.e., 4, 6, 8, and 12 h). This argument is further supported by our current observation that the ultradian behavioral cycle is entrained twice a day to align with the diurnal cycle. This could indicate that ultradian rhythms have evolved to go through several complete cycles within the day to allow synchronization to the circadian clock at least once every day, which may have an adaptive benefit as it could lock complementary phases of two different cycles together.

Whichever the mechanism is that drives these ultradian rhythms, they represent a novel class of intrinsic, non‐food intake‐driven ultradian rhythms. The largest cluster (*N* = 93) describes 3.4 h rhythms in lipid metabolites. Lipids have been reported to exhibit circadian oscillations in *Per1/2* double knockout mice that do not harbor functional circadian clocks and as a result also do not express circadian feeding rhythms,^50^ supporting our current observation of non‐circadian‐clock‐driven dynamics in lipid metabolism. The same study reported that feeding cycles could phase shift these non‐circadian‐clock‐driven rhythms, and indeed feeding rats on an enforced 6‐meal ultradian schedule also lead to changes in lipid metabolism.^51^ This finding suggests that lipid metabolism expresses rhythms with varied periods, covering both circadian and ultradian rhythms.

The cluster expressing 4.1 h ultradian rhythmicity is enriched for biogenic amines and encompasses 14 of the 21 detected amino acids. Ornithine (and arginine, although not deemed ultradian in this study) are directly implicated in the latter steps of the urea cycle, suggesting ultradian rhythms in liver urea formation. While our observations are made in the vole, arginine blood concentrations have also been shown to exhibit ultradian rhythms in healthy human male volunteers, and have been linked to insulin‐mediated glucose homeostasis.^52^ Indeed, both insulin and glucose have been shown to exhibit an ultradian pattern in blood concentrations in humans that persists during constant enteral nutrition^53^, ^54^ confirming intrinsic, non‐food‐driven ultradian rhythms in glucose homeostasis. Recently it has been suggested that other amino acids such as leucine and glutamine may be modulators of pancreatic insulin secretion,^55^ and both amino acids exhibit ultradian rhythmicity in our current metabolomics analysis. The observation of ultradian rhythms in amino acids, and the known ultradian patterns in human glucose and insulin levels provide evidence that these ultradian rhythms in amino acids are central to intrinsically driven ultradian glucose homeostasis.

Our observation of ultradian rhythms in amino acid metabolism also aligns with observations of intrinsically driven ultradian rhythms in cell mass in in vitro cell culture, which was linked to protein synthesis.^56^ Recently, it was also shown that a 4‐h rhythm in dry cell mass can be halted using proteostasis inhibitors,^22^ which is in line with the 4.1 h rhythm in amino acid concentration in this study.

### 4.4 Ultradian metabolic rhythm with a ~12 h period

A single cluster of metabolites exhibited an 11.9‐h ultradian rhythm, and no co‐expressed diurnal rhythm was detected in these metabolites. Ultradian rhythms with 12‐h period have regularly been reported in transcriptomics studies,^16^, ^17^, ^42^ and are sometimes attributed to result from intersecting circadian clocks, which has not been confirmed in clock‐less models. Our behavioral data suggest that a 12‐h rhythm can result from the intersection of ultradian and circadian rhythms, rather than two circadian rhythms. Indeed, it has been reported that 12‐h rhythms in mouse liver lipid metabolism rely on the presence of a circadian clock,^57^ which is also in line with the hypothesis that 12‐h ultradian rhythmicity may result from the intersection of rapid ultradian rhythms and circadian clocks.

The 11.9‐h rhythms are exclusively expressed in 11 PC ae metabolites (*ether‐linked phosphatidylcholines*). Ether lipids are major components of cell membranes, and can act as antioxidants, and dysregulation of these lipids is associated with health issues including cancers and metabolic disease.^58^ Moreover, a recent mouse liver microarray study focusing on 12‐h transcriptional rhythms identified that there was an enrichment in genes involved in glycerophospholipid and sphingolipid metabolism, which were hypothesized to be involved in ER/Golgi membrane homeostasis,^42^ but the driving mechanisms remain unknown.

## 5 CONCLUSIONS

Ultradian rhythms are often overlooked due to an insufficient sampling resolution in most protocols, and because they are masked by the co‐expressed diurnal or circadian rhythms in the same measures. To overcome these limitations, we used a novel design that maps behavioral activity/feeding state to high temporal resolution metabolic measures in voles, a model in which circadian dominance is reduced by ultradian feeding patterns. Our results describe clear interactions between diurnal and ultradian timing in behavior, including food intake. Critically, our results in the vole provide evidence for ultradian rhythms in circulating metabolites which do not result from postprandial excursions in metabolite concentration after ultradian feeding bouts, but appear to be intrinsically driven rhythms that are co‐expressed with diurnal rhythms in the metabolome. The observed interactions between ultradian and circadian behaviors, including feeding, and circulating metabolite rhythms fuel the need to recognize ultradian rhythms in voles and other species when studying the effects of mistimed eating by adopting high‐resolution sampling of metabolic physiology, and establishing the effects on phase and visibility of ultradian metabolic processing.

## AUTHOR CONTRIBUTIONS

Conceptualization: Raphaelle Winsky‐Sommerer, Menno P. Gerkema, Daan R. van der Veen. Methodology: Debra J. Skene, Menno P. Gerkema, Daan R. van der Veen. Investigation: Andreas Psomas, Namrata R. Chowdhury, Benita Middleton, Daan R. van der Veen. Supervision: Raphaelle Winsky‐Sommerer, Daan R. van der Veen. Formal Analysis: Andreas Psomas, Benita Middleton, Menno P. Gerkema, Daan R. van der Veen. Visualization: Andreas Psomas, Daan R. van der Veen. Writing—Original Draft Preparation: Andreas Psomas, Daan R. van der Veen. Writing—Review & Editing: Raphaelle Winsky‐Sommerer, Debra J. Skene, Menno P. Gerkema, Daan R. van der Veen.

## DISCLOSURES

The authors declare that they have no competing interests.

## Supporting information


Figure S1

Table S1

## Data Availability

The dataset supporting the conclusions of this article will be made available upon acceptance for publication.
