# Supplementary material for: Co‐expression of diurnal and ultradian rhythms in the plasma metabolome of common voles (Microtus arvalis)
Source: FASEB J. 2023 Mar 1;37(4):e22827. doi: 10.1096/fj.202201585R (PMC11977602; doi:10.1096/fj.202201585R)
Supplement: Supplementary file 1 — Figure S1 [file FSB2-37-e22827-s001.docx]

**Figure S1**


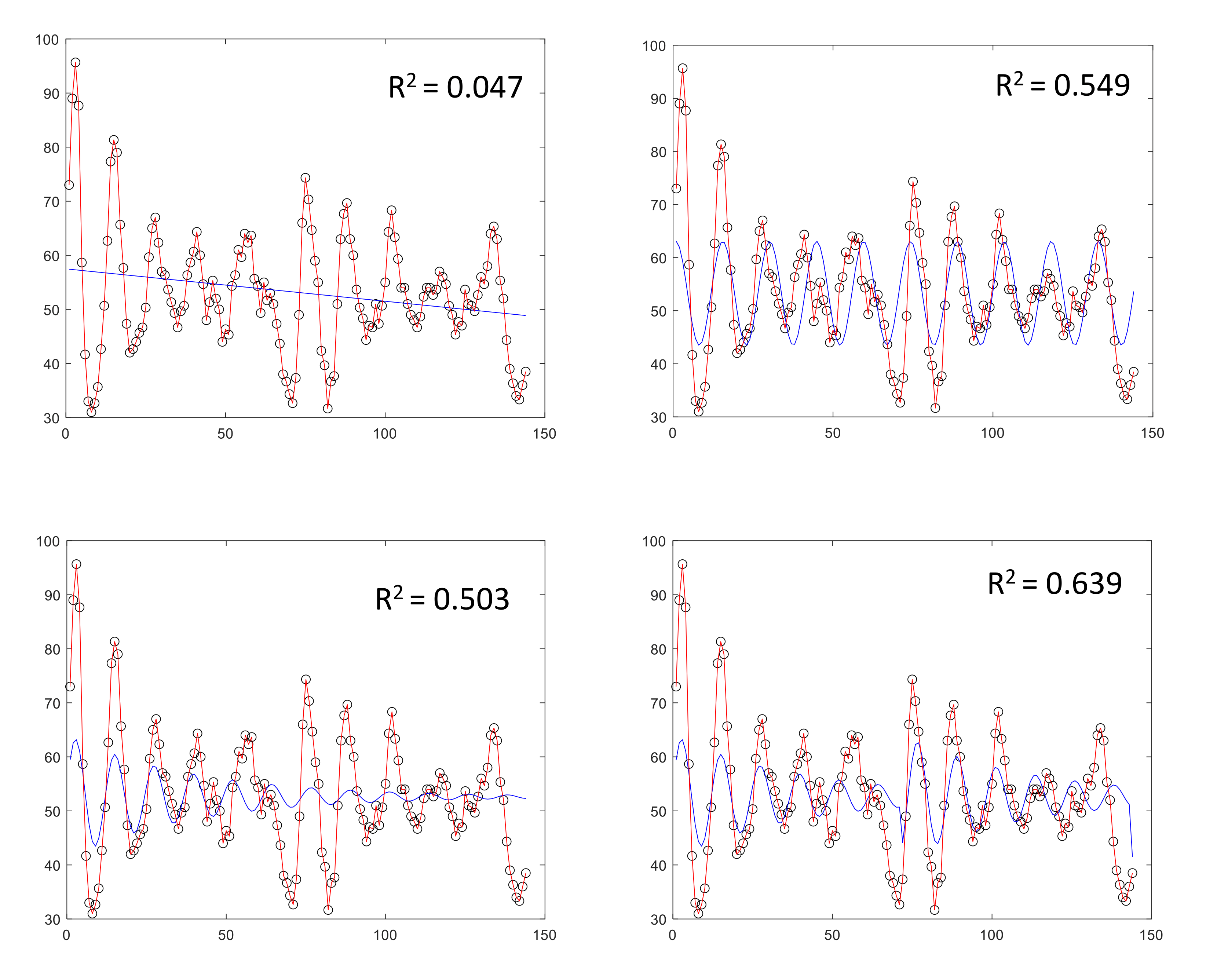


Cosine model fitting to histogram of onsets over the 24-hour light-dark cycle. Models used were a sloping line, standard cosine, dampening cosine and dampening cosine in which dampening is reset after 12 hours. Models are shown left-right, top to bottom. The last model, in which the dampening was reset after 12 hours exhibit the largest R^2^ of 0.639. Red line connects XY points, blue line is fitted model.
