# Supplementary material for: Co‐expression of diurnal and ultradian rhythms in the plasma metabolome of common voles (Microtus arvalis)
Source: FASEB J. 2023 Mar 1;37(4):e22827. doi: 10.1096/fj.202201585R (PMC11977602; doi:10.1096/fj.202201585R)
Supplement: Supplementary file 2 — Table S1 [file FSB2-37-e22827-s002.docx]

**Table S1:** Metabolites in the largest clusters expressing ultradian rhythms

| **Cluster I** |  | **Cluster V** |  |  |
| --- | --- | --- | --- | --- |
| Alanine |  | Phosphatidylcholine ae C36:4 |  |  |
| Asparagine |  | Phosphatidylcholine ae C36:5 |  |  |
| Glutamine |  | Phosphatidylcholine ae C38:4 |  |  |
| Glycine |  | Phosphatidylcholine ae C38:5 |  |  |
| Histidine |  | Phosphatidylcholine ae C38:6 |  |  |
| Isoleucine |  | Phosphatidylcholine ae C40:4 |  |  |
| Lysine |  | Phosphatidylcholine ae C40:5 |  |  |
| Methionine |  | Phosphatidylcholine ae C40:6 |  |  |
| Ornithine |  | Phosphatidylcholine ae C42:0 |  |  |
| Proline |  | Phosphatidylcholine ae C42:1 |  |  |
| Serine |  | Phosphatidylcholine ae C42:5 |  |  |
| Threonine |  |  |  |  |
| Tyrosine |  |  |  |  |
| Valine |  |  |  |  |
| Methionine-Sulfoxide |  |  |  |  |
| Sarcosine |  |  |  |  |
| Trans-4-Hydroxyproline |  |  |  |  |
| Carnitine |  |  |  |  |
| Propionylcarnitine |  |  |  |  |
|  |  |  |  |  |
| **Cluster II** | | | | |
| Leucine |  | Phosphatidylcholine aa C36:0 |  | Phosphatidylcholine ae C34:2 |
| Phenylalanine |  | Phosphatidylcholine aa C36:1 |  | Phosphatidylcholine ae C34:3 |
| Tryptophan |  | Phosphatidylcholine aa C36:2 |  | Phosphatidylcholine ae C36:0 |
| Kynurenine |  | Phosphatidylcholine aa C36:3 |  | Phosphatidylcholine ae C36:1 |
| Serotonin |  | Phosphatidylcholine aa C36:4 |  | Phosphatidylcholine ae C36:2 |
| Hydroxypropionylcarnitine |  | Phosphatidylcholine aa C36:5 |  | Phosphatidylcholine ae C36:3 |
| Propenylcarnitine |  | Phosphatidylcholine aa C36:6 |  | Phosphatidylcholine ae C38:0 |
| Lysophosphatidylcholine a C16:0 |  | Phosphatidylcholine aa C38:0 |  | Phosphatidylcholine ae C38:1 |
| Lysophosphatidylcholine a C16:1 |  | Phosphatidylcholine aa C38:3 |  | Phosphatidylcholine ae C38:2 |
| Lysophosphatidylcholine a C17:0 |  | Phosphatidylcholine aa C38:4 |  | Phosphatidylcholine ae C38:3 |
| Lysophosphatidylcholine a C18:0 |  | Phosphatidylcholine aa C38:5 |  | Phosphatidylcholine ae C40:1 |
| Lysophosphatidylcholine a C18:1 |  | Phosphatidylcholine aa C38:6 |  | Phosphatidylcholine ae C40:2 |
| Lysophosphatidylcholine a C18:2 |  | Phosphatidylcholine aa C40:1 |  | Phosphatidylcholine ae C40:3 |
| Lysophosphatidylcholine a C20:3 |  | Phosphatidylcholine aa C40:2 |  | Phosphatidylcholine ae C42:2 |
| Lysophosphatidylcholine a C20:4 |  | Phosphatidylcholine aa C40:3 |  | Phosphatidylcholine ae C42:3 |
| Lysophosphatidylcholine a C24:0 |  | Phosphatidylcholine aa C40:4 |  | Phosphatidylcholine ae C44:3 |
| Lysophosphatidylcholine a C26:0 |  | Phosphatidylcholine aa C40:5 |  | Phosphatidylcholine ae C44:4 |
| Lysophosphatidylcholine a C26:1 |  | Phosphatidylcholine aa C40:6 |  | Phosphatidylcholine ae C44:5 |
| Lysophosphatidylcholine a C28:0 |  | Phosphatidylcholine aa C42:0 |  | Phosphatidylcholine ae C44:6 |
| Lysophosphatidylcholine a C28:1 |  | Phosphatidylcholine aa C42:1 |  | Hydroxysphingomyelin C14:1 |
| Phosphatidylcholine aa C24:0 |  | Phosphatidylcholine aa C42:2 |  | Hydroxysphingomyelin C16:1 |
| Phosphatidylcholine aa C28:1 |  | Phosphatidylcholine aa C42:4 |  | Hydroxysphingomyelin C22:1 |
| Phosphatidylcholine aa C30:0 |  | Phosphatidylcholine aa C42:5 |  | Hydroxysphingomyelin C22:2 |
| Phosphatidylcholine aa C32:0 |  | Phosphatidylcholine aa C42:6 |  | Hydroxysphingomyelin C24:1 |
| Phosphatidylcholine aa C32:1 |  | Phosphatidylcholine ae C30:0 |  | Sphingomyelin C16:0 |
| Phosphatidylcholine aa C32:2 |  | Phosphatidylcholine ae C30:1 |  | Sphingomyelin C16:1 |
| Phosphatidylcholine aa C32:3 |  | Phosphatidylcholine ae C30:2 |  | Sphingomyelin C18:0 |
| Phosphatidylcholine aa C34:1 |  | Phosphatidylcholine ae C32:1 |  | Sphingomyelin C18:1 |
| Phosphatidylcholine aa C34:2 |  | Phosphatidylcholine ae C32:2 |  | Sphingomyelin C20:2 |
| Phosphatidylcholine aa C34:3 |  | Phosphatidylcholine ae C34:0 |  | Sphingomyelin C24:0 |
| Phosphatidylcholine aa C34:4 |  | Phosphatidylcholine ae C34:1 |  | Sphingomyelin C26:0 |
